# Physiological, Biochemical, and Metabolic Responses to Short and Prolonged Saline Stress in Two Cultivated Cardoon Genotypes

**DOI:** 10.3390/plants9050554

**Published:** 2020-04-27

**Authors:** Teresa Docimo, Rosalba De Stefano, Elisa Cappetta, Anna Lisa Piccinelli, Rita Celano, Monica De Palma, Marina Tucci

**Affiliations:** 1 Institute of Bioscience and BioResources, National Research Council, Via Università 100, 80055 Portici, Italy; 2 Department of Pharmacy, University of Salerno, Via Giovanni Paolo II 132, 84084 Fisciano, Italy

**Keywords:** salinity tolerance, phenylpropanoids, antioxidant enzymes, oxidative stress

## Abstract

Cultivated cardoon is a multipurpose crop with adaptability to limiting environments. Two genotypes (“Bianco Avorio” and “Spagnolo”) were comparatively characterized in response to short and prolonged 100 mM NaCl stress in hydroponics. Salt induced no growth variations between genotypes or symptoms of NaCl toxicity, but boosted ABA accumulation in roots and leaves. Both genotypes had high constitutive phenol content, whose major components were depleted upon 2 days of stress only in “Bianco Avorio”. Prolonged stress stimulated accumulation of proline, phenylpropanoids, and related transcripts, and non-enzymatic antioxidant activity. Decreased antioxidant enzymes activities upon short stress did not occur for APX in “Spagnolo”, indicating a stronger impairment of enzymatic defenses in “Bianco Avorio”. Nonetheless, H_2_O_2_ and lipid peroxidation did not increase under short and prolonged stress in both genotypes. Overall, the two genotypes appear to share similar defense mechanisms but, in the short term, “Bianco Avorio” depends mainly on non-enzymatic antioxidant phenylpropanoids for ROS scavenging, while “Spagnolo” maintains a larger arsenal of defenses. Upon prolonged stress, proline could have contributed to protection of metabolic functions in both genotypes. Our results provide cues that can be exploited for cardoon genetic improvement and highlight genotypic differences for breeding salinity tolerant varieties.

## 1. Introduction

Global warming intensifies soil salinity, severely impairing agricultural production. Excessive soil salinization is particularly increasing in arid and semi-arid cultivation areas, where evapotranspiration is more prominent than rainfall. In this scenario, salinity is becoming one of the most serious abiotic stresses affecting plant growth and development and hence crop productivity [1]. High salinity induces several major damages in plants. Osmotic stress due to water and nutrient uptake inhibition from roots and ion toxicity caused by excessive sodium entrance in the plant cell result in unbalanced photosynthesis and metabolism, which in turn produce a large amount of ROS (reactive oxygen species) and consequently oxidative stress. Based on their adaptive mechanisms in saline habitats, plants have been divided into “halophytes” (salt-tolerant) and “glycophytes” (salt-sensitive), the former providing a model to elucidate key mechanisms of salt tolerance. To survive in high salinity conditions, halophytes developed effective salt tolerance through several abilities: 1) Control of the osmotic movement of water in and out of the cytoplasm of the cells to prevent dehydration; 2) enhanced exclusion and sequestration of excess salts to adjust water potential; 3) regulation of Na^+^ and Cl^−^ uptake while maintaining cytoplasmic K^+^ and Mg^2+^ at the necessary concentrations for ensuring enzyme functions; and 4) synthesis of organic solutes to allow ROS scavenging [2,3,4,5]. Although tolerance mechanisms are ubiquitous, according to [2,6,7] major differences between tolerant and sensitive plants can be associated to signal perception and processing, resulting from genetic variability of a complex trait. In the perception process, the phytohormone ABA (abscissic acid) plays an important role in triggering a wide array of metabolic and physiological changes towards adaptation to saline environment. Among others, the production of osmolytes as well as enzymatic and non-enzymatic antioxidants, such as phenylpropanoids, are the strategy adopted by numerous plants to limit oxidative stress caused by salinity [3,8]. There is a general consensus indicating that antioxidant enzymes might constitute the first line of defense against ROS damage [9]. Nevertheless, severe stress conditions can negatively affect the enzymatic machinery [10,11]. Polyphenols, which are often the major secondary metabolites of plant leaves, constitute the core unit of the non-enzymatic antioxidant machinery. These molecules, mostly the dihydroxy benzene derivatives, have been shown in several plants to assist the action of antioxidant enzymes, especially when multiple stresses are occurring [12,13]. Phenylpropanoids are ubiquitous in plants and, differently from other secondary metabolites and enzymes, are not all compartmentalized in plant cells. Therefore they can perform a complementary protective (antioxidant) function in an organelle-independent manner [13,14]. Moreover, whereas activities of antioxidant enzymes are often dependent on variables such as light and temperature [11], phenylpropanoids are more constant and therefore might play a prominent defensive role in severe stress conditions that strongly reduce enzyme performances.

Hence, tolerance derives from a tight and efficient interaction between biochemical signals, which act in both additive and synergistic ways in the plant response to salinity. In this regard, investigation of mechanisms of salt resistance/tolerance has been pursued in several species for the development of salt-tolerant genotypes, thus opening the possibility to use salinized land for crop production.

*Cynara cardunculus* L. var *altilis*, the cultivated cardoon, is a perennial species native to the Mediterranean region. Cardoon requires low nutrients and nitrogen and is usually cultivated in arid and semi-arid environments, where soil salinity can be remarkable. Several studies highlighted the highly adaptive potential of cardoon, focusing on its ability to face abiotic stressors such as salt, drought and heavy metals [15,16,17,18,19,20,21]. Being able to resist environmental constraints while maintaining a high production of biomass, cardoon has gained interest as a multipurpose crop with a wide array of applications. Cardoon leaves, for their high content in natural antioxidants can be used either for food and for nutraceutical and pharmaceutical applications [22,23]. Along with some phenylpropanoids, cardoon produces other specialized metabolites, i.e., sesquiterpenes lactones (STL), which are peculiar to most Asteraceae [24]. Cynaropicrin is the major STL found in Cynaroideae and confers a distinctive flavor, mostly associated to the bitter taste of the edible parts of cardoon and globe artichoke [25,26]. Besides its organoleptic properties, cynaropicrin, as many other STLs, has beneficial activities in plant-environment interaction and human health protection [27,28]. In a biorefinery perspective, cardoon biomass provides cellulose for paper industry and lignocellulosic feedstock for energy and green chemistry, while valuable oil with many non-food applications is recovered from the seeds. Thus cardoon cultivation can provide the combined production of biofuels (e.g., bioethanol) and bio-based products [29,30] in an environmental and energetically efficient way.

References [19,31] reported how this species overcomes salt stress by acting as a facultative halophyte. These authors [31] showed that cardoon was significantly inhibited by moderately high concentrations of 100 mM NaCl or KCl and, similarly to halophytic plants, perceived high concentrations of Na^+^ as less toxic than K^+^ and, conversely to most of the glycophytes, counteracted osmotic unbalance through regulation of inorganic ion content, translocating Na^+^ excess to the shoots. Most probably, cardoon is able to tolerate Na^+^ through a tissue tolerance mechanism, although this species might use also other mechanisms to overcome saline stress. In accordance, more recently [19] showed that, under NaCl imposition, cardoon modifies its water uptake capability by reducing seedlings water content, and this mechanism can be considered an adaptive strategy.

Studies on the potential of Italian commercial cardoon genotypes focused on production of biomass, energy or other valuable products, also with very low agronomical inputs [32,33,34]. Besides, the potential of cardoon cultivation to produce bioactive compounds has also been evaluated. Reference [17] reported that a number of cultivated genotypes, including “Bianco Avorio”, can accumulate higher amounts of flavonoids compared to other wild and cultivated genotypes in a hydroponic floating system upon saline elicitation. Another study reported that “Spagnolo” had the best detoxification mechanisms under metal stress conditions [16].

Since “Bianco Avorio” and “Spagnolo” appear very promising and particularly suited for cultivation under low input management in temperate climates, we characterized these cardoon genotypes for both their performances under severe saline stress conditions and tolerance mechanisms. In the present work, the physiological, transcriptional, enzymatic and metabolic changes determined by short and prolonged NaCl stress imposition on the two cardoon genotypes “Bianco Avorio” and “Spagnolo” were analyzed in order to assess the traits mostly involved in saline tolerance. Our study highlights the metabolic investment of cardoon plants to face salt stress and provides new evidence on how cardoon plants use their antioxidant machinery to prevent oxidative damage caused by growth in saline environments. Overall, this study delivers new findings about cardoon saline tolerance and may supply useful cues in designing strategies for breeding improved genotypes for sustainable cultivation.

## 2. Results

### 2.1. Morphological Traits

Salinity tolerance is commonly related to plant’s capability of maintaining growth under stressful conditions in limiting environments. In this study, evaluation of plant growth of two cardoon genotypes, “Bianco Avorio” and “Spagnolo”, indicated that both genotypes are tolerant to our prolonged saline stress conditions, i.e., 100 mM NaCl treatment (12.0 mS cm^−1^) in hydroponic conditions for up to 21 days (Table 1). Measurement of the last leaf width highlighted an invariance of this parameter, a proxy of leaf area, between salt treated and untreated (0 mM NaCl, 1.92 mS cm^−1^) plants of both genotypes. Shoot biomass showed no significant differences between the two genotypes and in comparison with their respective controls in terms of dry weight, though a significant decrease in shoot fresh weight was detected in both genotypes. Stimulation of root growth was detected in both genotypes, with an increase of specific root length of about 4% in “Bianco Avorio” and 12% in “Spagnolo” under NaCl stress (Table 1).

During the time frame of our hydroponic experiment on the effects of NaCl salinity on cardoon, we did not detect symptoms of ion toxicity or senescence, such as leaf dropping or yellowing for either of the two genotypes (Figure S1). Chlorophyll content measurements according to the mean SPAD index recorded similar values in control conditions and after NaCl treatment in both genotypes, namely no significant differences were detected between genotypes and treatments (Table 2).

### 2.2. Effect of Salt Stress on ABA Concentration

**Figure 1:**
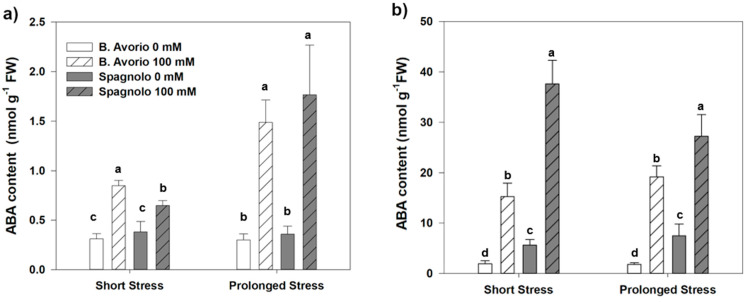
Levels of abscisic acid (ABA) in (**a**) roots and (**b**) leaves of “Bianco Avorio” and “Spagnolo” *C. cardunculus* var *altilis* genotypes after 2 and 21 days of 0 (control) and 100 mM (salinity treatment) NaCl. Each value represents the mean ± SD of three biological replicates. Values indicated with different letters are significantly different between genotypes and treatments within the same sampling time at *p* < 0.05, by analysis of variance (ANOVA).

The phytohormone ABA is known to mediate environmental cues by regulating metabolic and transcriptional networks that modulate plant growth and development and accumulates at high levels in response to stresses such as drought and salinity. In order to get a deeper insight in the cardoon response to salt, we followed the ABA accumulation in roots and leaves of the two genotypes “Bianco Avorio” and “Spagnolo” during the 100 mM NaCl treatment (Figure 1a,b). Short and prolonged salt stress significantly induced ABA production in both organs and cardoon genotypes. In roots, ABA increased to a slightly higher level in “Bianco Avorio” than in “Spagnolo” after 2 days of NaCl treatment, whereas after 21 days no significant differences emerged between the genotypes, both revealing an ABA induction almost four times higher than the control level (Figure 1a). In leaves, “Bianco Avorio” maintained a steady increase of ABA levels (4–5-fold above controls) with increasing time of NaCl exposure, whereas “Spagnolo” accumulated high levels of ABA after 2 days of NaCl stress, but this accumulation slight declined upon prolonged stress (Figure 1b).

### 2.3. Metabolites Production under Salt Stress

**Figure 2:**
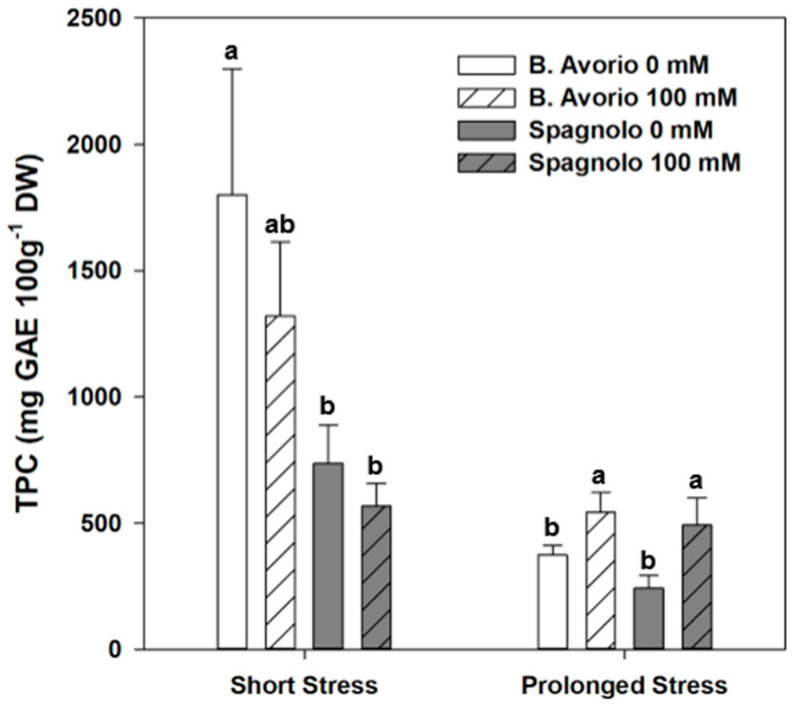
Total phenolics content (TPC) in leaves of “Bianco Avorio” and “Spagnolo” genotypes of *C. cardunculus* var *altilis* after 2 and 21 days of 0 (control) and 100 mM (salinity treatment) NaCl, expressed as gallic acid equivalents (GAE) and measured by the ABTS assay. Each value represents the mean ± SD of three biological replicates. Values indicated with different letters are significantly different between genotypes and treatments within the same sampling time at *p* < 0.05, by analysis of variance (ANOVA).

In several species, alleviation of salt-induced oxidative stress by the accumulation of specific metabolites, especially phenolic acids, contributes to increasing plant resistance. In order to reveal possible physiological strategies behind the cardoon response to high NaCl, the non-enzymatic antioxidant machinery was investigated in the two genotypes, both upon short and prolonged salt stress. The total phenolic content (TPC) of leaves (Figure 2), evaluated by the Folin Ciocalteau assay, indicated high levels of phenolic compounds at 2 days for both genotypes (1.8–1.3 and 0.6–0.7 g GAE/100 g DW in “Bianco Avorio” and “Spagnolo”, respectively). However, no significant difference between control and short saline stress was observed. After 21 days, the TPC values in control conditions were reduced (0.4 and 0.2 g GAE/100 g DW in “Bianco Avorio” and “Spagnolo”, respectively), but they significantly increased above control levels upon salt stress (about 0.5 g GAE/100 g DW in “Bianco Avorio” and “Spagnolo”) (Figure 2).

Reference [17] reported the effect of mild salt treatment on phenylpropanoids yield in several cardoon genotypes, including “Bianco Avorio”. Thus, based on the TPC results and literature data, we further investigated the dynamics of leaf phenylpropanoid accumulation and its relation to NaCl stress tolerance upon short and prolonged salt stress by UHPLC profiling of the cardoon leaf. Identified compounds were phenolic compounds belonging to cinnamylquinic acids (chlorogenic acid, 5-CQA; *p*-coumaroylquinic acid, pCoQA; feruloylquinic acid, FQA), dicaffeoylquinic acids (1,3-, 3,4-, 1,5-diCQA; 3,5- and 4,5-diCQA), and flavones (luteolin-rutinoside, LRUT; luteolin-glucoside, LGLU; luteolin-glucuronide, LGLA; luteolin-malonylglucoside, LmGLU; luteolin, L; and apigenin, A) [35,36,37] and the sesquiterpene lactone cynaropicrin [25] (Table S2). Namely, in both cardoon genotypes, the major constituents of leaves were chlorogenic acid (5-CQA), 1,5-diCQA and 3,5-diCQA, along with luteolin (L) and its glycoside derivatives (Lgly) (Table 3). Mirroring TPC data, at 2 days control leaves produced large amounts of caffeoylquinic acids and flavones (Table 3). “Bianco Avorio” showed the highest levels, with about 3 and 0.6 g 100 g^−1^ DW of caffeoylquinic derivatives (CQAs) and flavones, respectively, whereas in “Spagnolo” they were about 4 times less abundant. Upon 2 days of saline stress a depletion (about 50%) of phenylpropanoids and flavones was observed in “Bianco Avorio”, whereas in “Spagnolo” leaves the level of CQAs remained comparable to controls. After 21 days, a strong reduction of both phenolic classes was detected in unstressed leaves of the two investigated genotypes (level of total phenols < 0.02 g 100 g^−1^ DW). However, the long-term saline stress induced a significant increase in their amount in both genotypes (CQAs: *p* < 0.01; Ls < 0.05). In detail, at 21 days of saline treatment, the caffeoylquinic acids pool increased more than thirty times above the control in both genotypes, though slightly more in “Bianco Avorio”. Similarly, a significant increase in the levels of luteolin and its glycosides was detected in both genotypes.

In order to investigate whether other peculiar Asteraceae metabolites could be affected by saline stress in the two cardoon genotypes, the levels of the sesquiterpene lactone cynaropicrin in control and salt stress conditions at the two sampling times were compared by UHPLC analysis (Figure S2). Data indicated no significant changes in “Bianco Avorio” unstressed or stressed leaves at 2 or 21 days of NaCl treatment, whereas a slight increase was detected only in “Spagnolo” upon 21 days of salt imposition.

### 2.4. Transcriptional Changes in Phenylpropanoids and Sesquiterpenes Biosynthetic Genes

The relationship of transcriptional abundance of key phenylpropanoid and sesquiterpenes biosynthetic genes with metabolic changes within each genotype and treatment was further investigated. Namely, variation in the transcriptional activities was monitored at 21 days of saline stress imposition, when the largest metabolic changes were observed. We examined expression levels of Hydroxycinnamoyl transferase *HCT*, hydroxy cinnamoyl quinate transferase *HQT*, Flavonoid synthase *FNSII*, Flavonoid 3′Hydroxylase, *F3*′*H* and of the transcription factor (TF) *MYB12*, known as regulator of phenylpropanoid biosynthesis, in cardoon unstressed and 21 days NaCl-stressed leaves. Alongside, we investigated key genes involved in sesquiterpene biosynthesis, namely germacrene A synthase *GAS*, germacrene A oxidase *GAO* and custonolide synthase *COS*.

**Figure 3:**
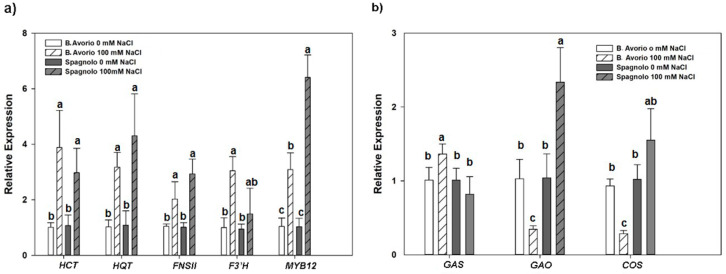
Effects of salt stress on relative expression of phenylpropanoids and sesquiterpenes key biosynthetic genes measured upon prolonged (21 days) stress treatment (100 mM NaCl) in leaves of “Bianco Avorio” and “Spagnolo” genotypes of *C. cardunculus* var *altilis*. (**a**) qRT- PCR analysis of *HCT, HQT, FNSII*, *F3*′*H* and *MYB12* TF; (**b**) qRT-PCR analysis of *GAS, GAO* and *COS*. Results are expressed as fold changes relatively to the relevant unstressed control (21 days, 0 mM NaCl) of each genotype. Each value represents the mean ± SD of three biological and 2 technical replicates. Values indicated with different letters are significantly different between genotypes and treatments at *p* < 0.05, by analysis of variance (ANOVA).

Expression of the genes coding for committed enzymes in CGA and flavonoids biosynthesis, namely *HCT HQT* and *FNSII, F3*′*H*, respectively, were upregulated by NaCl stress in both genotypes to a similar extent. A significant accumulation of MYB12 transcripts was detected both in “Bianco Avorio” and “Spagnolo” stressed leaves, although significantly higher in “Spagnolo” (Figure 3a). Regarding the expression of sesquiterpene lactone biosynthetic committed genes, responsible for the formation of cynaropicrin, the level of the GAS transcription was slightly triggered by NaCl only in “Bianco Avorio”, whereas *GAO* and *COS* showed a contrasting trend between the genotypes, with a significant decrease in “Bianco Avorio” and a significant increase in “Spagnolo” (Figure 3b). The latter result is consistent with the higher production of cynaropicrin in “Spagnolo” after prolonged stress (Figure S2).

### 2.5. Physiological Traits: Non-Enzymatic Antioxidant Machinery

**Figure 4:**
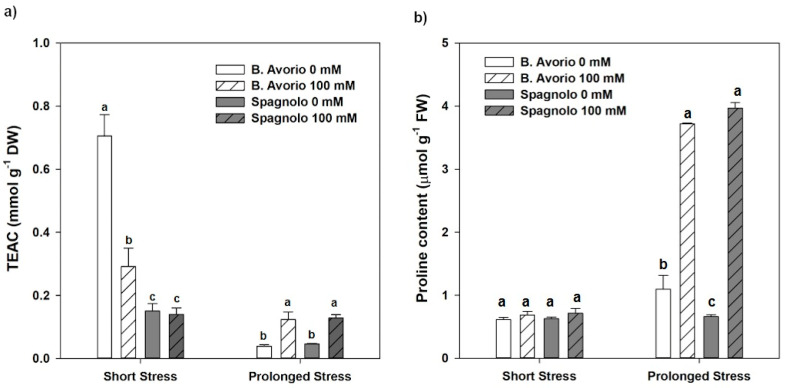
(**a**) Antioxidant activity, measured by ABTS assay and expressed as Trolox equivalent antioxidant capacity (TEAC), and (**b**) proline content in leaves of “Bianco Avorio” and “Spagnolo” genotypes of *C. cardunculus* var *altilis* after 2 and 21 days of 0 (control) and 100 mM (salinity treatment) NaCl. Each value represents the mean ± SD of three biological replicates. Values indicated with different letters are significantly different between genotypes and treatments within the same sampling time at *p* < 0.05, by analysis of variance (ANOVA).

The trend observed for TP was reflected by the changes in antioxidant capacity (Figure 4a). In the short term, a higher antioxidant power was detected by the ABTS (2,2′-azino-bis (3-ethylbenzothiazoline-6-sulfonic acid)) assay in “Bianco Avorio” leaf extracts compared to “Spagnolo” in control conditions (0.7 vs. 0.2 mmol TEAC g^−1^ DW). Upon NaCl stress, no changes were detected in “Spagnolo”, while a significant reduction to about 0.3 mmol TEAC g^−1^ DW was observed in “Bianco Avorio”, whose antioxidant capacity was nonetheless significantly higher than “Spagnolo”. After 21 days, the antioxidant capacity of control leaves was much lower than after two days and was not different between genotypes, but it increased almost three times upon salt stress in both genotypes. As a non-enzymatic antioxidant, we measured proline content in leaves during short and prolonged stress (Figure 4b). At 2 days, proline levels were similar in control and stressed leaves, regardless of the genotype. Prolonged salt stress induced a strong accumulation of proline, with an almost three (≈3.8 µmol g^−1^ FW) and six (≈4 µmol g^−1^ FW) times increase respect to controls in “Bianco Avorio” and “Spagnolo” leaves, respectively.

### 2.6. Enzymatic Antioxidant Machinery

To maintain metabolic efficiency and hence normal growth and development, a balanced ratio between production and detoxification of oxygen radicals is necessary, and more so under stress. Therefore, antioxidant enzymes are often reported to play an important role in imparting tolerance in plants under saline conditions. To investigate the ROS scavenging ability of “Bianco Avorio” and “Spagnolo”, we measured the activities of superoxide dismutase SOD, catalase CAT, and ascorbate peroxidase APX, during short and prolonged NaCl stress (Table 4). Upon 2 days of saline stress, the enzyme activities were significantly reduced compared to controls in “Bianco Avorio”, whereas in “Spagnolo” CAT and SOD showed the same trend, except for APX, whose enzymatic activity showed a significant increase. Under prolonged saline stress (21 days), the activity of CAT recovered in both genotypes and was not different from the controls, while SOD and APX activities significantly declined.

### 2.7. Oxidative Stress Detection

**Figure 5:**
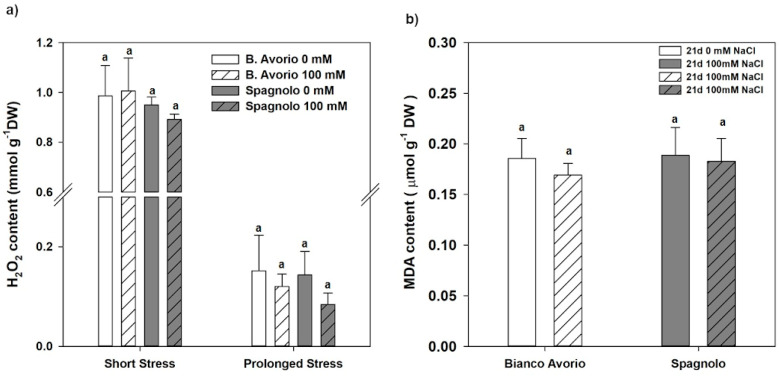
(**a**) H_2_O_2_ content measured in leaves of “Bianco Avorio” and “Spagnolo” genotypes of *C. cardunculus* var *altilis* after 2 and 21 days of 0 (control) and 100 mM (salinity treatment) NaCl; (**b**) MDA content in leaves “Bianco Avorio” and “Spagnolo” genotypes of *C. cardunculus* var *altilis* measured upon prolonged salt stress. Each value represents the mean ± SD of three biological replicates. Values indicated with different letters are significantly different between genotypes and treatments within the same sampling time at *p* < 0.05, by analysis of variance (ANOVA).

In order to verify whether cardoon genotypes were able to counteract the negative effects of reactive oxygen species (ROS) produced under stress conditions, we measured malonyldialdeide MDA and hydrogen peroxide H_2_O_2_ production in “Bianco Avorio” and “Spagnolo” leaves (Figure 5a,b). Both assays revealed that the two genotypes did not show oxidative damage under prolonged NaCl stress conditions (21 days; Figure 5a). Namely, H_2_0_2_ content was not significantly different from the controls in both genotypes. Similarly, the evaluation of the oxidative status marker MDA revealed no differences between control and stressed leaves in both genotypes.

## 3. Discussion

Cultivation in saline soils or irrigation with low quality saline water, instead of the increasingly demanded fresh water, can contribute to a more sustainable agriculture. For these practices to become more widely diffuse, it is necessary to evaluate the effects of saline stress on plant growth and development as well as to elucidate the mechanisms of salinity tolerance/sensitivity, in order to design breeding strategies for salt resistant varieties. Cardoon is classified as a species well adapted to moderately high salt concentration [31]. Nevertheless, NaCl treatment has been reported to negatively affect biomass production of several wild and cultivated cardoon genotypes except for “Bianco Avorio”, which accumulated higher biomass under salinity [17]. Moreover, the genotype “Spagnolo” showed better morphological and physiological characters than “Sardo” and “Siciliano” when tested on metal contaminated soil [16]. Given the potential of “Bianco Avorio” and “Spagnolo” as biomass and multipurpose genotypes in limiting environments, we evaluated their growth responses and stress tolerance mechanisms under short and prolonged NaCl treatment. In our experimental conditions, prolonged exposure to salt (100 mM NaCl for 21 days) did not compromise aerial growth of both genotypes in terms of dry biomass production. Further, the absence in both genotypes of dead leaves and of a significant reduction of new tissue growth, as well as invariance of the fourth leaf width, support a minor effect of salt on shoot growth, which commonly affects leaf area [38]. According to these data, we might exclude that the two cardoon genotypes suffered from either osmotic or ionic toxicity, since translocation of Na^+^ excess to older leaves would have caused leaf yellowing and/or dropping and lack of photosynthates exportation to young leaves would have determined growth reduction of new leaves [39]. The invariance of the chlorophyll index under NaCl treatment in “Spagnolo” confirms earlier results on the maintenance of the photosynthetic apparatus in this genotype in metal-contaminated soil [16]. Indeed, as reported for other species [40], we detected an increase of root growth, indicated by root DW and higher root/shoot ratio (R/S, below-/above-ground dry mass in this study). Changes of the R/S index may differ according to plant species and stress intensity [41]. An increase in R/S, i.e., a comparatively higher biomass allocation in roots than shoots, can result in accumulation of toxic ions in roots and hence minimize their negative effects on shoot growth when shoots are more sensitive to the stressful conditions [42]. In another interpretation, the increase of R/S might be a strategy to cope with stress through increased root exploration in search of NaCl-free soil [43]. Overall, the observed growth responses confirmed the good performance of “Bianco Avorio” observed by [17], even at the higher NaCl concentration tested in this study (100 mM vs. 30 mM), and indicate that also “Spagnolo” tolerates NaCl stress.

Plant perception of a stress status, mainly water deficit and salt excess, results in ABA biosynthesis [2], which in turn is responsible for biochemical, physiological and morphological changes, especially regarding root growth. Our data indicate that ABA accumulation triggered by salt perception in roots increases over time with almost comparable levels in both genotypes, whereas in leaves it is higher in “Spagnolo” than “Bianco Avorio” throughout the salt exposure period. This increase in root ABA levels may support higher root elongation observed in both genotypes, which was significant only in “Spagnolo”, similarly to the increased total root length induced by ABA in salt-stressed tomato [44].

Secondary metabolites, especially phenylpropanoids, accumulate in plants in order to counteract oxidative damage induced by abiotic stressors [45] Our metabolic data indicate that both cardoon genotypes have high TPC (Figure 2) and high levels of chlorogenic acid conjugates and flavonoids, which were the major TPC components as demonstrated by HPLC quantitative analysis at two days in control conditions, being almost 4 times higher in “Bianco Avorio” than “Spagnolo” (Table 3). Short saline treatment reduced TPC and significantly depleted the amount of total CQAs only in “Bianco Avorio”, suggesting that this genotype uses these metabolic “defensive” reservoirs against oxidative stress under short salt treatment. The metabolic behavior of “Bianco Avorio” appears to develop in two phases, with a fast depletion of antioxidants, most probably to promptly face stress injuries, in the early response to salt stress, followed by accumulation of phenylpropanoids for maintaining growth and ROS homeostasis in the second phase (prolonged stress). On the contrary, “Spagnolo” does not significantly recruit these antioxidants upon short exposure to NACl stress, but eventually engages them upon prolonged stress (Figure 2 and Table 3). The same occurs for the major cardoon flavonoids, such as the flavone luteolin and its glycoside conjugate (Table 3), in either of the two genotypes. Biosynthesis of antioxidant flavonols has been reported to be induced by several abiotic stresses in rice and Arabidopsis [46,47] to take advantage of their special ability to scavenge ROS through their B dihydroxylated rings [11,13,48]. Accumulation of flavonoids may also have a role in auxin movement within and between cells, thus maintaining cellular growth and plant development [49]. Noteworthy, the highest level of TPC and of their major cardoon components was observed after prolonged stress, when we also detected the highest ABA accumulation, in accordance with several studies reporting that ABA modifies some classes of secondary metabolites, especially phenylpropanoids [50]. The observed changes in phenylpropanoids levels in the two genotypes in response to short and prolonged NaCl stress are reflected by the changes in non-enzymatic antioxidant capacity (Figure 4). Indeed, strong antioxidant defenses may have contributed to the tolerance to heavy metals observed in “Spagnolo” by [16]. In “Spagnolo” prolonged salinity determined a slight increase in cynaropicrin (Figure S2). Several authors reported cynaropicrin accumulation in cardoon and artichoke as localized in specific tissues and mostly dependent on developmental stages, genotype, and harvesting time [26,51]. Moreover, in other Asteraceae high de novo production of STLs in leaves or their release from glandular trichomes is usually associated to a feeding attack from chewing insects or birds, indicating that a repelling flavor can be a protective mechanism [28]. To our knowledge, this metabolite is not reported to contribute to abiotic stress tolerance as it does for biotic stresses [27,52]. Nonetheless, although not investigated in this work, we cannot exclude a contribution of methylerythritol phosphate pathway precursors to stress tolerance in “Spagnolo” [11]. Moreover, we cannot exclude that the growth conditions and plant age in our experiments could have been unfavorable to a stronger cynaropicrin induction.

Expression profiling of biosynthetic and regulatory genes involved in phenylpropanoids and sesquiterpenes formation at 21 days of salt stress, when most metabolic changes occurred, supported the metabolic trends. Salt stress significantly increased the expression levels of phenylpropanoid-related genes and of Myb12 TF in both genotypes. This MYB TF is a regulator of flavonol production [53] and its stress induced expression, especially in “Spagnolo”, might be related to the recruitment of flavonoids as powerful antioxidants [46,47,48]. Changes in the expression level of cynaropicrin biosynthetic genes also mirrored cynaropicrin accumulation in “Spagnolo” at 21 days of stress, with the key biosynthetic genes in sesquiterpenes formation GAO and COS showing a significant increase of transcriptional abundance (Figure 3b and Figure S2). Overall, these results indicate that salinity orchestrates transcriptional and metabolic fluxes in a genotype-dependent manner.

Although different plants may use individual strategies to fine-tune defensive responses under stress, quite often there is a close relationship between proline accumulation and stress tolerance. Accumulation of compatible solutes (e.g., proline, raffinose, and glycine betaine) is a common mechanism for plants to cope with stress by maintaining cellular turgor [2]. In addition, it is also known that compatible solutes can help to stabilize proteins and cellular structures and to counteract oxidative stress. In both “Bianco Avorio” and “Spagnolo” leaf proline strongly increased upon prolonged stress, possibly contributing to maintain the integrity of photosynthetic apparatus and membranes under saline stress, as indicated by invariant chlorophyll content (Table 2) and lack of lipid peroxidation (Figure 5b). These data suggest that this osmolyte might actively participate to protective mechanisms of metabolic adaptation under salinity in cardoon. However, its role as antioxidant [54] cannot be proved from our data, since changes in proline levels in response to NaCl exposure (2 and 21 days) in the two cardoon genotypes do not parallel the changes in non-enzymatic antioxidant activity (Figure 4), which on the contrary reflects mostly, if not totally, phenylpropanoids accumulation in relation to stress level and duration, as well as genotype.

It is reported in several species [55,56] that salt stress strongly reduces antioxidant enzymatic activities and that, under severe stress, SOD and CAT activities seem to be inversely correlated with phenylpropanoid biosynthesis. In cardoon, the main antioxidant enzymatic activities, i.e., SOD, CAT, and APX significantly declined under short saline treatment in “Bianco Avorio”, whereas in “Spagnolo” APX activity was enhanced. Instead, prolonged NaCl imposition exerted similar effects in both genotypes, with a decline of activities of SOD and APX, while CAT resumed to control levels (Table 4). These results suggest that short salinity stress strongly reduced the antioxidant enzymatic activities in both genotypes, but to a lower extent in “Spagnolo”, as shown by the induction of APX, thus possibly indicating a minor damage to the enzymatic machinery in this genotype. The increase of this detoxifying activity might explain why “Spagnolo” did not show a significant recruitment of antioxidant phenylpropanoids in reaction to short stress compared to “Bianco Avorio”. Upon prolonged stress, partial inactivation of antioxidant enzymes could be compensated by the increase in phenylpropanoids biosynthesis observed in both genotypes. Additionally, we found that two markers of oxidative stress, i.e., MDA, derived from ROS-damaged membranes, and H_2_O_2_, were not different in NaCl-treated leaves compared to their respective controls, confirming that both “Bianco Avorio” and “Spagnolo” were able to tolerate the stress levels applied in this study.

Although the results need to be tested in field conditions, where other factors such as soil pH may have significant effects, they corroborate the idea that severe NaCl conditions did not impair growth and development of “Bianco Avorio” and “Spagnolo” and that phenolic antioxidants and proline more than antioxidant enzymes could serve as main tolerance mechanism in these cardoon genotypes. Albeit we report that both genotypes are tolerant to NaCl treatment, however “Spagnolo” is able to better tolerate stress by maintaining the integrity of crucial biochemical functions that could impart defensive advantages on the long term.

## 4. Materials and Methods

### 4.1. Experimental Design

Seeds of *Cynara cardunculus* L. var. *altilis* cv. “Bianco Avorio” and “Spagnolo”, kindly provided by La Semiorto Sementi, Lavorate di Sarno, Italy) and Arca 2010 (Acerra, Italy), respectively, were germinated in soil and transplanted after one month into hydroponics containers into a temperature-controlled greenhouse of the CNR-IBBR Portici (27 °C day/24 °C night). Each experimental unit consisted of 48 plants of each genotype per treatment in a completely randomized block design. Plants were kept in half-strength modified Hoagland nutrient solution (1.23 mS cm^−1^ electrical conductivity, EC) for about 7 days, followed by 7 additional days in full-strength modified nutrient solution (electric conductivity of 1.92 mS cm^−1^) as described by [40,57]. Each container was filled with 10.0 L of aerated nutrient solution. The obtained plants for each genotype were then randomly divided between control and NaCl treatment. The saline treatment started forty-five days post sowing. For the 100 mM NaCl treatment, salt was added stepwise in two consecutive days up to 100 mM (electric conductivity of 12.0 mS cm^−1^) and maintained for 21 days. The pH of the nutrient solutions was 6.0 ± 0.3. All nutrient solutions were prepared using analytical grade chemicals and deionized water. To prevent large fluctuations in EC, pH, and ionic concentrations, the nutrient solutions were completely renewed from all tanks weekly. Within each treatment and genotype, 16 randomly selected plants were harvested for short saline stress measurements at 2 days of 100 mM NaCl imposition, and 16 plants were used for prolonged stress measurements after 21 day of 100 mM NaCl imposition. The remaining 16 plants per thesis (genotype × treatment) were used for destructive biometric parameters analysis, as below described.

For RNA extraction and biochemical analyses, for each genotype, treatment and time point, four biological replicates consisting of four plants were collected. Leaves and roots were harvested in liquid nitrogen and frozen at −80 °C.

### 4.2. Growth Parameters

For growth evaluation, 16 cardoon plants for each genotype and treatment were harvested at 21 days, recording the width of the youngest fully-expanded leaf (last leaf), and shoot and roots fresh weight. Shoot and root tissues were then dried in a forced-air oven at 60 °C for 72 h for dry biomass determination (DW). Root DW/shoot DW ratio (R:S) and specific root length, corresponding to the total root length/root DW (SRL) were measured according to [58]

Leaf greenness was measured using a portable chlorophyll meter soil plant analysis development (SPAD) (SPAD 502 Plus Meter, Konica Minolta, Japan) in plants exposed to 2 and 21 days of 0 mM (control) or 100 mM NaCl (stress treatment) treatment. SPAD readings were the average of 3 measurements from at the central point of the leaf between the midrib and the leaf margin of the third leaf starting from the top. For each genotype/treatment, SPAD values are expressed as mean ± SD of ten plants.

### 4.3. ABA Determination

For ABA extraction, freeze-dried root and leaf samples (150 mg) were homogenized in liquid nitrogen and incubated in 2 mL of water overnight at 4 °C. After sample centrifugation at 9300× *g* for 10 min, quantitative free ABA determination was performed on three biological triplicates and technical duplicates by the competitive ELISA Phytodetek ABA test kit (Agdia Incorporated, San Diego, IN, USA) following the provider’s protocol. Color absorbance was read at 405 nm using a plate auto-reader (1420 Multilabel Counter Victor3TM, PerkinElmer, Boston, MA, USA) and free ABA content was expressed in nmol g^−1^ FW.

### 4.4. Analysis of Secondary Metabolites

#### 4.4.1. Extraction

Exhaustive extraction of the cardoon leaf phenolic fraction was performed according to [35]. In detail, dried leaf samples were ground and then extracted by ultrasound-assisted solid–liquid extraction, for 30 min (×4) at 25 °C and at 20.0 kHz in a thermostat-controlled ultrasound bath (Labsonic LBS2, Falac Instruments, Treviglio, Italy), using aqueous methanol (80% *v*/*v*) and a matrix/solvent ratio of 1:20. The pooled extracts were dried under a gentle nitrogen flow. Finally, the residues were reconstituted with 1 mL of aqueous methanol (50% *v*/*v*) and filtered through 0.20 μM regenerated cellulose filters (CHROMAFIL Xtra RC-20/13, Delchimica, Milano, Italy), before the chromatographic analysis. Dilutions of the extracts were used for determination of total phenol content and for ABTS assays, as reported below.

#### 4.4.2. Determination of Total Phenolics Content (TPC)

TPC of cardoon leaves was determined using the Folin–Ciocalteu colorimetric method according to [59]. Briefly, 20 μL of diluted leaf extract and 5 μL of Folin–Ciocalteu reagent were added to 145 μL of ultrapure water in a 96-well microplate. Then 30 μL of Na_2_CO_3_ (20%, *w*/*v*) were added in each well and the reaction mixtures were incubated at 25 °C for 45 min. Absorbances were read at 725 nm with a Multiskan Go microplate spectrophotometer (Thermo Fisher Scientific, Walthaman, MA, USA). Gallic acid (GA) was used as reference standard and TPC was estimated from the GA calibration curve (range 5–200 µg/mL, 7 seven levels; R^2^ = 0.999). The results were expressed as GA equivalents per 100 g of dried leaf (mg GAE 100 g^−1^ DW, means ± standard deviation of three biological replicates).

#### 4.4.3. Secondary Metabolite Profiling by UHPLC-DAD-HRMS

To determine the qualitative profile of cardoon metabolites, the characterization of leaf extracts was performed by UHPLC-DAD-HRMS. Analyses were carried out using a PLATINblue UHPLC system (Knauer, Labservice Analytica, Berlin, Germany) equipped with a Kinetex C18 column (2.1 × 100 mm, 2.6 mm; Phenomenex, Bologna, Italy) and connected to a LTQ OrbiTrap XL mass spectrometer (Thermo Fisher Scientific, Walthaman, MA, USA) operating in negative ionization mode with an ESI source. The chromatographic and mass spectrometer conditions were the same as those reported by [35]. Metabolite assignments were made comparing retention time, UV and mass spectra, accurate mass and characteristic fragmentation of detected compounds with standard compounds, whenever available, or interpreting MS data (accurate masses and MS/MS fragment ions) combined with chemo-taxonomic data reported in the literature and databases. The Xcalibur software (version 2.2, Thermo Fisher Scientific, Walthaman, MA, USA) was used for instrument control, data acquisition, and data analysis.

#### 4.4.4. Quantitative Analysis of Target Polyphenols and Cynaropicrin

5-CQA, 1,5- and 3,5-diCQA, luteolin glycosides (Lgly, sum of LRUT, LGLU, and LGLA), luteolin and cynaropicrin were detected in significant quantities in all the different leaf extracts. Their contents in extracts were determined by UHPLC-UV (5-CQA, 1,5- and 3,5-diCQA and cynaropicrin) and UHPLC-MS (luteolin glycosides) analyses. The chromatographic conditions were the same as those used for UHPLC-DAD-HRMS analysis. In the UHPLC-UV analysis, the chromatograms were recorded at 325, 350, and 220 nm for the detection of caffeoylquinic acids, flavones, and cynaropicrin, respectively. External standard calibration method was used to quantify the main phenolic components of cardoon leaves (5-CQA, 1,5-, and 3,5-diCQA), according to previously validated method [35]. Mixtures of reference standards, at different concentrations, were used to produce calibration curves (range of 0.5–10 μg mL^−1^). For diCQAs (1,5- and 3,5-diCQA), 1,3-diCQA was used as reference standard. Cynaropicrin levels in leaf samples were estimated related to UV response. The levels of luteolin and its glycoside derivatives was estimated using MS detection in MRM mode (LRUT, *m*/*z* 593 → 285; LGLU, *m*/*z* 447 → 285; LGLA, m/z 461 → 285; L, *m*/*z* 285 → 151), due to their low and variable levels in the different samples that not allowed the UV quantification. Luteolin glycosides amounts were calculated using calibration curve of luteolin-7-*O*-glucoside. The amount of the compounds was finally expressed as mg per 100 g of dried leaf (mg 100 g^−1^ DW, mean ± standard deviation of three biological replicates).

### 4.5. RNA Extraction and qRT-PCR

Leaves of cardoon plants exposed to 2 days and 21 days of 0 or 100 mM NaCl were collected for RNA extraction and biochemical analyses. Samples were ground in liquid nitrogen and total RNA was extracted from 100 mg of tissue using the RNeasy kit (Qiagen, Germantown, MD, USA). After the visual check of RNA integrity on agarose gels and quantification using a NanoDrop ND-8000 spectrophotometer (Thermo Fisher Scientific, Walthaman, MA, USA), 1 μg of total RNA was reversed transcribed using the QuantiTec Reverse Transcription Kit (Qiagen, Germantown, MD, USA), according to the manufacturer’s instructions. Real-time qRT-PCR was performed with Platinum^®^ SYBR^®^ Green qPCR Super Mix (Thermo Fisher Scientific, Walthaman, MA, SA) in a ABI7900 HT (Thermo Fisher Scientific, Walthaman, MA, USA). Each PCR reaction (20 μL) contained 10 μL real-time qRT-PCR Mix, 4 μL of a 1:25 dilution of cDNA and 0.25 μM of each specific primer designed on sequences retrieved either from X. The thermal cycling conditions were 50 °C for 2 min, 95 °C for 2 min, followed by 40 cycles of 15 s at 95 °C and 30 s at 60 °C. PCR product melting curves were analyzed for the presence of a single peak. All reactions were performed on biological triplicates and technical duplicates and fold change measurements calculated with the 2^−ΔΔCT^ method [60,61]. Each genotype in control condition was used as internal calibrator. Gene expression was normalized on the stably expressed cardoon actin gene. Sequences of primers used for real-time qRT-PCR are provided in Table S1.

### 4.6. Non-Enzymatic Antioxidants: ABTS and Proline

The ABTS scavenging capacity assay was carried out in 96-well plates according to [59]. Briefly, 500 μL of ABTS^•+^ solution (about 1 mM) was mixed with 5 μL of diluted extracts (five dilutions with approximately 20–80% control absorbance), Trolox (2–25 μM, 6 levels) reference standard solutions or PBS (control), and 300 μL of the mixtures were transferred into a 96-well plate. The absorbance was measured at 734 nm, after 60 min of incubation in the dark at 30 °C, with a microplate spectrophotometer reader Multiskan Go (Thermo Fisher Scientific, Walthaman, MA, USA). Trolox was used as reference standard (range 2–25 µM, 6 levels, R^2^ = 0.999). ABTS assay results were expressed as TEAC per 100 mg DW of extract (μmoL TE 100 g^−1^ DW, mean ± standard deviation of three biological replicates).

For Proline determination, fresh leaf samples (250 mg) were homogenized in liquid nitrogen and extracted with 70:30 ethanol:water mixture (*v*/*v*), then 50 µL of extracts were mixed with 100 µL of acidic ninhydrin reagent (ninhydrin 1% (*w*/*v*) in acetic acid 60% (*v*/*v*), ethanol 20% (*v*/*v*)), following a procedure previously described by [59]. Proline concentration was determined from a standard curve and calculated on a fresh weight basis (µmol Proline g^−1^ FW) using three biological and three technical replicates.

### 4.7. Antioxidant Enzymes

Antioxidant enzyme activities were measured in leaves sampled at 2 days and 21 days, as previously reported. Fresh leaf material (100 mg) was rapidly frozen in liquid nitrogen and extracted with 0.5 mL of 100 mM potassium phosphate buffer (pH 7.0) containing ethylenediamine tetraacetic acid (EDTA). The extracts were then centrifuged at 11,000× *g* at 4 °C for 15 min, and the supernatant used for all assays. Preparation of the enzyme extracts was carried out at 0–4 °C. An aliquot of the extract was used to determine spectrophotometrically protein content, reading absorption of protein extracts at 590 nm based on bovine serum albumin (BSA) standard curve (in the range 0.25–2 mg ml^−1^) and expressed in mg g^−1^ FW according to Bradford assay [62]. Spectrophotometric measurements of enzyme activities were performed on Multiskan Sky UV-VIS Spectrophotometer (Thermo Fischer Scientific, Walthaman, MA, USA). Superoxide dismutase (SOD; EC 1.15.1.1) activity was measured at 560 nm, with the commercial kit OxiSelect™ Superoxide Dismutase Activity Assay (Cell Biolabs Inc., San Diego, CA, USA) according to the manufacturer’s instructions. SOD enzyme activity was expressed as SOD units mg^−1^ protein. Catalase (CAT, EC 1.11.1.6) activity was measured at 240 nm, determining the rate of conversion of H_2_O_2_ to O_2_ and water, as described by [63]. CAT enzyme activity was expressed as µmol H_2_O_2_ min^−1^ mg^−1^ protein. Ascorbate peroxidase (APX, EC 1.11.1.11) activity was determined following the H_2_O_2_-dependent oxidation of ascorbic acid (ASA) at 265 nm in a reaction mixture composed of 50 mM ASA, 90 mM H_2_O_2_, 20 µL protein extract and 0.1 M phosphate buffer (pH 6.4) [64]. The activity of APX was corrected by subtracting the non-enzymatic H_2_O_2_-dependent ASA oxidation and the H_2_O_2_-independent ASA oxidation. APX activity was expressed as µmol ASA min^−1^ mg^−1^ protein. All the assays were performed in three biological and two technical replicates in 96 well plates and were adapted to small volumes. The amount of proteins was calculated.

### 4.8. MDA and H_2_O_2_ Assays

Lipid peroxidation was estimated by determining the malonyldialdehyde (MDA) contents in leaves. One hundred milligrams of dried samples were homogenized in 2 mL of 0.1% TCA. The homogenate was centrifuged at 15,000× *g* for 10 min at 4 °C. A 0.5 mL aliquot of the supernatant was mixed with 1.5 mL of 0.5% TBA prepared in TCA 20% and incubated at 90 °C for 20 min. After stopping the reaction in an ice bath, samples were centrifuged at 10,000× *g* for 5 min. The supernatant absorbance at 532 nm was then measured. After subtracting the non-specific absorbance at 600 nm, MDA concentration (three replicates per treatment) was determined using the extinction coefficient 155 mM^−1^ cm^−1^. H_2_O_2_ was determined by measuring potassium iodide (KI) oxidation by H_2_O_2_ in acidic medium according to [65]. Cardoon leaf tissues (100 mg) were homogenized in ice bath with 1 mL 0.1% (*w*/*v*) TCA. The homogenate was centrifuged at 12,000× *g* for 15 min and 0.25 mL of the supernatant was added to 0.25 mL 10 mM potassium phosphate buffer (pH 7.0) and 0.5 mL 1 M KI. The absorbency of supernatant was read at 390 nm. A calibration curve obtained with H_2_O_2_ standard solutions prepared in 0.1% TCA was used for quantification

### 4.9. Statistical Analysis

Results are presented as mean ± standard deviation (SD). The statistical significance of data was evaluated through one-way analysis of variance (ANOVA). Statistical significance was set at *p* < 0.05 and the Tukey’s post hoc test was applied for mean separation. SigmaStat version 11.0 software was used to perform statistical comparisons.

## Figures and Tables

**Table 1:** Growth-related parameters of “Bianco Avorio” and “Spagnolo” genotypes of *Cynara cardunculus* var *altilis* under control (0 mM NaCl) and stress (100 mM NaCl) conditions at the end of the stress period (21 days).

Growth Parameters	NaCl mM	“Bianco Avorio”	“Spagnolo”
**last leaf width** mm	0	47.44 ± 6.61 ^a^	42.45 ± 6.56 ^a^
	100	43.17 ± 4.16 ^a^	46.49 ± 5.00 ^a^
**shoot FW** g plant^−1^	0	22.35 ± 6.74 ^a^	22.28 ± 6.74 ^a^
	100	15.02 ± 4.41 ^b^	15.00 ± 5.11 ^b^
**shoot DW** g plant^−1^	0	1.37 ± 0.47 ^a^	1.27 ± 0.44 ^a^
	100	1.07 ± 0.32 ^a^	1.15 ± 0.49 ^a^
**root FW** g plant^−1^	0	3.19 ± 1.52 ^b^	3.52 ± 1.24 ^b^
	100	4.34 ± 1.26 ^ab^	5.50 ± 1.82 ^a^
**root DW** g plant^−1^	0	0.26 ± 0.18 ^b^	0.27 ± 0.15 ^b^
	100	0.39 ± 0.16 ^ab^	0.46 ± 0.29 ^a^
**root length** cm	0	22.2 ± 6.58 ^b^	25.59 ± 7.86 ^b^
	100	27.31 ± 8.36 ^ab^	37.81±14.55 ^a^
**R:S**	0	0.22 ± 0.19 ^b^	0.23 ± 0.11 ^b^
	100	0.34 ± 0.13 ^a^	0.39 ± 0.14 ^a^
**SRL** cm g^−1^	0	82.5 ± 33.3 ^a^	86.5 ± 36.4 ^a^
	100	86.5 ± 41.1 ^a^	97.5 ± 41.3 ^a^

Values are expressed as mean of biological replicates (n = 8) ± SD. Values followed by different superscript letters are significantly different between genotypes and treatments at the *p* < 0.05, by analysis of variance (ANOVA).

**Table 2:** Chlorophyll content (SPAD value) of the two cultivated *C. cardunculus* var *altilis* genotypes “Bianco Avorio” and “Spagnolo” after 0, 2, and 21 days of 0 (control) and 100 mM (salinity treatment) NaCl.

Time (day)	“Bianco Avorio”		“Spagnolo”	
	0 mM NaCl	100 mM NaCl	0 mM	100 mM NaCl
0	22.45 ± 5.62 ^a^	25.16 ± 6.25 ^a^	23.13 ± 3.62 ^a^	23.66 ± 4.99 ^a^
2	20.47 ± 4.61 ^a^	20.75 ± 4.70 ^a^	23.39 ± 2.72 ^a^	24.73 ± 3.87 ^a^
21	26.88 ± 6.70 ^a^	32.42 ± 5.95 ^a^	29.77 ± 7.55 ^a^	26.28 ± 6.82 ^a^

Values are expressed as mean of biological replicates (n = 10) ± SD. Values followed by different superscript letters are significantly different between genotypes and treatments within the same time at the *p* < 0.05, by analysis of variance (ANOVA).

**Table 3:** Polyphenolic content in leaves of “Bianco Avorio” and “Spagnolo” *C. cardunculus* var *altilis* genotypes after 2 and 21 days of 0 (control) and 100 mM (salinity treatment) NaCl.

Time (Day)	Genotypes	NaCl (mM)	CQA Tot	5-CQA	diCQAs	Lgly	L
			mg/100 g DW				
2	Bianco Avorio	0	2861.0 ± 753 ^a^	893.0 ± 68.8 ^a^	1968.0 ± 794.0 ^a^	540.3 ± 163.0 ^a^	8.2 ± 3.0 ^a^
		100	1502.3 ± 351.5 ^b^	599.3 ± 202.8 ^b^	903.1 ± 311.0 ^ab^	308.8 ± 53.0 ^b^	6.0 ± 2.1 ^ab^
	Spagnolo	0	698.3 ± 202.5 ^c^	346.2 ± 133.3 ^c^	352.0 ± 128.3 ^b^	113.2 ± 31.5 ^c^	2.0 ± 0.1 ^c^
		100	578.2 ± 84.1 ^c^	220.0 ± 51.8 ^c^	358.2 ± 36.7 ^b^	50.0 ± 2.8 ^d^	3.1 ± 0.2 ^bc^
21	Bianco Avorio	0	8.2 ± 2.6 ^b^	4.5 ± 0.9 ^b^	3.6 ± 1.8 ^b^	0.8 ± 0.1 ^b^	2.3 ± 1.4 ^b^
		100	285.7 ± 61.9 ^a^	156.2 ± 46.0 ^a^	129.5 ± 28.2 ^a^	17.2 ± 1.9 ^a^	9.0 ± 3.0 ^a^
	Spagnolo	0	8.9 ± 1.4 ^b^	4.8 ± 0.6 ^b^	4.1 ± 1.8 ^b^	3.5 ± 1.9 ^b^	4.3 ± 0.9 ^b^
		100	248.9 ± 62.1 ^a^	83.0 ± 28.2 ^a^	165.9 ± 34.7 ^a^	57.9 ± 25.9 ^a^	13.4 ± 5.8 ^a^

5-CQA (caffeoylquinic derivatives): chlorogenic acid; diCQAs: dicaffeoylquinic acids (sum of 1,5- and 3,5-dicaffeoylquinic acid); CQA tot: sum of caffeoylquinic derivatives and chlorogenic acids; Lgly: luteolin glycosides; L: luteolin. Each value represents the mean ± SD of three biological replicates. Values followed by different superscript letters are significantly different between genotypes and treatments within each sampling time, according to one way ANOVA (*p* < 0.05).

**Table 4:** SOD, CAT, and APX activities in leaves of “Bianco Avorio” and “Spagnolo” genotypes of *C. cardunculus* var *altilis* after 2 and 21 days of 0 (control) and 100 mM (salinity treatment) NaCl.

Time (Days)	Genotypes	NaCl (mM)	SOD (U mg^−1^ Protein)	CAT µmol H_2_O_2_ min^−1^ mg^−1^ Protein	APX µmol ASA min^−1^ mg^−1^ Protein
2	Bianco Avorio	0	571.7 ± 81.8 ^a^	0.54 ± 0.05 ^a^	9.30 ± 1.52 ^a^
		100	253.6 ± 9.8 ^b^	0.31 ± 0.14 ^b^	2.26 ± 0.90 ^b^
	Spagnolo	0	236.9 ± 29.6 ^a^	0.37 ± 0.06 ^a^	3.58 ± 1.54 ^b^
		100	200.7 ± 15.3 ^b^	0.24 ± 0.05 ^b^	6.46 ± 0.92 ^a^
21	Bianco Avorio	0	358.4 ± 68.9 ^a^	0.39 ± 0.03 ^a^	3.21 ± 1.04 ^a^
		100	279.3 ± 23.6 ^b^	0.35 ± 0.05 ^a^	1.79 ± 0.21 ^b^
	Spagnolo	0	383.5 ± 25.2 ^a^	0.34 ± 0.02 ^a^	2.71 ± 0.29 ^a^
		100	258.6 ± 60.6 ^b^	0.31 ± 0.05 ^a^	1.62 ± 0.87 ^b^

Each value represents the mean ± SD of three biological replicates. Values followed by different superscript letters are significantly different between genotypes and treatments within the same sampling time at *p* < 0.05, by analysis of variance (ANOVA).

## Supplementary Materials

The following are available online at https://www.mdpi.com/2223-7747/9/5/554/s1, Table S1: List of primers used for qRT- PCR analysis in this study. Table S2: (‒)-UHPLC-HRMS/MS data of compounds detected in cardoon leaves. Figure S1: Phenotype of “Bianco Avorio” and “Spagnolo” C. cardunculus var altilis genotypes at 21 days of hydroponic cultivation in 0 mM NaCl and 100 mM NaCl nutrient solutions, Figure S2: UHPLC-UV analysis of cynaropicrin accumulation in plants of the “Bianco Avorio” and “Spagnolo” C. cardunculus var altilis genotypes after 2 and 21 days of hydroponic cultivation in control (0 mM NaCl) and 100 mM NaCl nutrient solutions. Each value represents the mean ± SD of three biological replicates. Different superscript letters indicate significant differences between genotypes and treatments within each sampling time, according to one way ANOVA (*p* < 0.05).
