# Supplementary material for: Physiological, Biochemical, and Metabolic Responses to Short and Prolonged Saline Stress in Two Cultivated Cardoon Genotypes
Source: Plants (Basel). 2020 Apr 27;9(5):554. doi: 10.3390/plants9050554 (PMC7284779; doi:10.3390/plants9050554)
Supplement: Supplementary file 1 [file plants-09-00554-s001.pdf]

Physiological, biochemical and metabolic responses to short and prolonged saline stress in two cultivated cardoon genotypes

Teresa Docimo *[1], Rosalba De Stefano [1], Elisa Cappetta [1], Anna Lisa Piccinelli [2], Rita Celano [2], Monica De Palma [1], Marina Tucci *[1].

[1] Institute of Bioscience and BioResources, National Research Council, Via Università 100, 80055 Portici (NA), Italy

Teresa.docimo@ibbr.cnr.it, (T.D.) destefano.rosalba@libero.it, (R. D.S) elisa.cappetta@ibbr.cnr.it, (E.C) monica.depalma@ibbr.cnr.it, (M. D.P.) marina.tucci@ibbr.cnr.it (M.T.)

[2] Department of Pharmacy, University of Salerno, Via Giovanni Paolo II 132, 84084 Fisciano (SA), Italy

apiccinelli@unisa.it, (A.L. P) rcelano@unisa.it (R. C.)

***Correspondence:** teresa.docimo@ibbr.cnr.it, marina.tucci@ibbr.cnr.it.

Supplementary Material

Table S1 List of primers used for qRT- PCR analysis in this study

Gene name	Function	Accession	Primer sequence
HCT	Hydroxycinnamoyl-CoA:shikimate hydroxycinnamoyltransferase	DQ104740	F5GCTAACACGAGACCAAGTCAATGCA<br>R5CACCGCCAAACATGACCAGAGA
HQT	Hydroxycinnamoyl-CoA quinate transferase	DQ915590	F5-TCA CAC AGG TTA CAC GCT TCA ACT G<br>R5-GGG CTT TAT CGG ACC ATG TAT TGA T
F3'H	Flavonoid 3′-hydroxylase	HM153534	F5 CCTGCAAAGCGTGACGAAGA<br>F3 ACGACGTCACCGCCATTTTT
FNSII	Flavone synthase	JN825735	F5 CGGCTGCAACGGATACAACA<br>F3 CTCGCGATTTCGAGCACCTT
MYB12	MYB Transcription factor	MG517449	F5TGAGGTTTGAAGGTGATGACACGC<br>R5GCCTTTTTTGCACTTTCCACACTC
GAS	Germacrene A synthase	JN383985	F5-CAAGACGTTTGGTGTGTCGG-3<br>R5-TCTCTTGGCTTGAGACACCC-3
GAO	Germacrene A oxidase	KF752449	F5-GCCCTGAGTTCCCATTGACA-3<br>R5-TCAGTTCGGAAATCGCCCAT-3
COS	Costunolide synthase	KF752452	F5-AATCGTAAACGCCTGGGCA-3<br>R5-TGAACTCGAAGTCTGCACCC-3
ACTIN	Actin	XM_025103545	F5-TACTTTCTACAACGAGCTTC-3<br>R5-ACATGATTTGAGTCATCTTC-3

Table S2 (–)-UHPLC-HRMS/MS data of compounds detected in cardoon leaves

.N	tR (min)	$[M–H]^–$ (m/z)	Molecular Formula	ppm	Diagnostic product ions (m/z)	Compound[a]	Ref.
1	4.2	353.0857	$C_{16}H_{18}O_9$	– 2.9	191.0543, $C_7H_{11}O_6^–$ (– 3.7 ppm); 179.0331, $C_9H_7O_4^–$ (– 4.5 ppm)	chlorogenic acid [a]	-
2	5.4	337.0909	$C_{16}H_{18}O_8$	– 2.7	191.0542, $C_7H_{11}O_6^–$ (– 4.2 ppm); 163.0383, $C_9H_7O_3^–$ (– 3.7 ppm)	p-coumaroylquinic acid	1
3	6.0	367.1012	$C_{17}H_{20}O_9$	– 3.2	193.0490, $C_{10}H_9O_4^–$ (– 2.6 ppm); 191.0544, $C_7H_{11}O_6^–$ (– 3.1 ppm)	feruloylquinic acid	1
4	7.4	515.1177	$C_{25}H_{24}O_{12}$	– 1.5	353.0850, $C_{16}H_{17}O_9^–$ (– 4.8 ppm); 191.0542, $C_7H_{11}O_6^–$ (– 4.2 ppm)	1,3-diCQA	2
5	8.7	593.1466	$C_{27}H_{30}O_{15}$	– 5.8	285.0385 ($C_{15}H_9O_6$, – 3.1 ppm)	luteolin-O-rutinoside	2
6	9.0	447.0905	$C_{21}H_{20}O_{11}$	– 3.8	285.0382 ($C_{15}H_9O_6$, – 3.9 ppm)	luteolin-7-O-glucoside [a]	-
7	9.2	461.0693	$C_{21}H_{18}O_{12}$	– 4.7	285.0382 ($C_{15}H_9O_6$, – 4.2 ppm)	luteolin-O-glucuronide	2
8	11.0	515.1165	$C_{25}H_{24}O_{12}$	– 3.6	353.0852, $C_{16}H_{17}O_9^–$ (– 4.2 ppm); 173.0436, $C_7H_9O_5^–$ (– 4.7 ppm)	3,4-diCQA	3
9	12.1	515.1160	$C_{25}H_{24}O_{12}$	– 4.7	353.0858, $C_{16}H_{17}O_9^–$ (– 2.5 ppm); 191.0542, $C_7H_{11}O_6^–$ (– 4.2 ppm)	1,5-diCQA	2
10	12.6	515.1157	$C_{25}H_{24}O_{12}$	– 5.2	353.0856, $C_{16}H_{17}O_9^–$ (– 3.1 ppm); 191.0543, $C_7H_{11}O_6^–$ (– 3.7 ppm)	3,5-diCQA	3
11	14.2	515.1168	$C_{25}H_{24}O_{12}$	– 3.0	353.0852, $C_{16}H_{17}O_9^–$ (– 4.3 ppm); 173.0436, $C_7H_9O_5^–$ (– 5.1 ppm)	4,5-diCQA	2
12	17.2	533.0897	$C_{24}H_{22}O_{14}$	– 5.4	489.1020, $[M–H–CO_2]^–$ (–1.5 ppm); 285.0380, $C_{15}H_9O_6$ (– 4.8 ppm)	luteolin-O-malonylglucoside	4
13	19.9	391.1374[b]	$C_{20}H_{24}O_8$	– 3.3	289.1065 $[C_{15}H_{16}O_3+HCOO]^–$ (–1.9 ppm); 101.0229 $C_4H_5O_3^–$ (–4.2 ppm)	cynaropicrin	*
14	20.4	285.0387	$C_{15}H_{10}O_6$	– 2.4	241.0489, $[M–H–CO_2]^–$ (–2.6 ppm); 151.0020, $^{1,3}A^–$ (– 4.0 ppm)	Luteolin [a]	-
15	21.7	269.0438	$C_{15}H_{10}O_5$	– 2.3	225.0539, $[M–H–CO_2]^–$ (– 3.2 ppm); 151.0019, $^{1,3}A^–$ (– 4.5 ppm)	Apigenin [a]	-

[a] Compared with reference standards; [b] m/z values corresponding to $[M+HCOOH–H]^–$;

References: 1. ref 37 in main manuscript; 2. Reference 35 in the manuscript; 3. Ref 36 in the manuscript; 4. Ref 59 in the manuscript.

*. Database mzCloud.

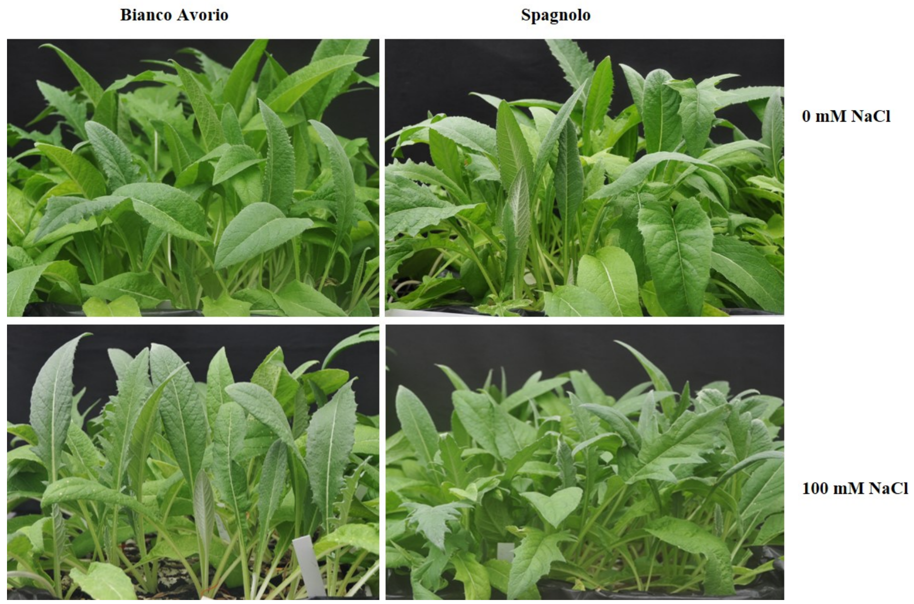

Figure S. 1 Phenotype of "Bianco Avorio" and "Spagnolo" *C. cardunculus var altilis* genotypes at 21 days [illegible] hydroponic cultivation in 0 mM NaCl and 100 mM NaCl nutrient solutions.

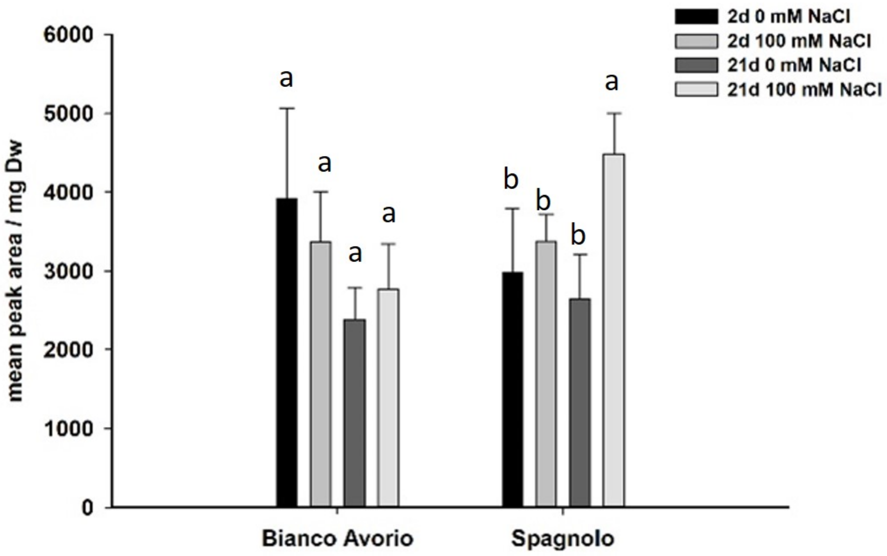

Figure S. 2. UHPLC-UV analysis of cynaropicrin accumulation in plants of the "Bianco Avorio" and "Spagnolo" *C. cardunculus var altilis* genotypes after 2 and 21 days of hydroponic cultivation in control (0 mM NaCl) and 100 mM NaCl nutrient solutions. Each value represents the mean ± SD of three biological replicates. Different superscript letters indicate significant differences between genotypes and treatments within each sampling time, according to one way ANOVA ($P < 0.05$).
